# Effects of Regular Classes in Outdoor Education Settings: A Systematic Review on Students’ Learning, Social and Health Dimensions

**DOI:** 10.3390/ijerph14050485

**Published:** 2017-05-05

**Authors:** Christoph Becker, Gabriele Lauterbach, Sarah Spengler, Ulrich Dettweiler, Filip Mess

**Affiliations:** 1 Department of Sports and Health Sciences, Technical University of Munich, Arcisstr. 21, 80333 Munich, Germany; gabriele.lauterbach@tum.de (G.L.); sarah.spengler@tum.de (S.S.); filip.mess@tum.de (F.M.); 2 Department of Cultural Studies and Languages, University of Stavanger, 4036 Stavanger, Norway; ulrich.dettweiler@uis.no

**Keywords:** outdoor education, school, children, adolescents, curriculum, social, learning, health, review

## Abstract

Background: Participants in Outdoor Education Programmes (OEPs) presumably benefit from these programmes in terms of their social and personal development, academic achievement and physical activity (PA). The aim of this systematic review was to identify studies about regular compulsory school- and curriculum-based OEPs, to categorise and evaluate reported outcomes, to assess the methodological quality, and to discuss possible benefits for students. Methods: We searched online databases to identify English- and German-language peer-reviewed journal articles that reported any outcomes on a student level. Two independent reviewers screened studies identified for eligibility and assessed the methodological quality. Results: Thirteen studies were included for analysis. Most studies used a case-study design, the average number of participants was moderate (mean valued (M) = 62.17; standard deviation (SD) = 64.12), and the methodological quality was moderate on average for qualitative studies (M = 0.52; SD = 0.11), and low on average for quantitative studies (M = 0.18; SD = 0.42). Eight studies described outcomes in terms of social dimensions, seven studies in learning dimensions and four studies were subsumed under additional outcomes, i.e., PA and health. Eleven studies reported positive, one study positive as well as negative, and one study reported negative effects. PA and mental health as outcomes were underrepresented. Conclusion: Tendencies were detected that regular compulsory school- and curriculum-based OEPs can promote students in respect of social, academic, physical and psychological dimensions. Very little is known concerning students’ PA or mental health. We recommend conducting more quasi-experimental design and longitudinal studies with a greater number of participants, and a high methodological quality to further investigate these tendencies.

## 1. Introduction

Within the past 20 years, Outdoor Education Programmes (OEPs) in general have been reported to show a number of positive effects on personal and social development, physical activity, academic achievement and leadership skills for a wide range of participants and age groups [1,2,3].

With a more specific focus on education within the school context, regular compulsory school-based and curriculum-based outdoor education programmes seem to have several positive effects on students’ physical activity levels [4], mental health status [5], social competences and relations [6,7], and academic achievement [8].

An all-encompassing definition of outdoor education is scarcely possible due to different meanings, understandings and practices within various research areas, countries and cultures [9]. Common terms include: learning outside the classroom, udeskole, friluftsliv, outdoor adventure education and forest school. In general, outdoor education can be described as teaching and/or learning and/or experiencing in an outdoor and/or out-of-school environment. The content of learning and teaching is therefore different and depends on the general aim of the programme, the target group and the outdoor setting, e.g., the gaining of knowledge in natural sciences; increased PA (physical activity), leadership skills, personal and social development; survival skills; and improved skills in relation to nature-oriented sports.

In contrast to these more general outdoor education programmes, we have concentrated on programmes that are embedded within the curriculum and are conducted regularly within the school schedule. These programmes focus on student-centred classes and interdisciplinary subjects, hands-on learning, possibilities to explore and experience oneself and the environment, and the use of natural and cultural places as a “classroom” [10,11]. Regular school-based and curriculum-based outdoor education programmes are still a rare phenomenon—with the exception of the grassroots movement of udeskole/uteskole in Scandinavia [12] which has increased during the last decade. It shows that, for example, 17.9% of all public schools and 19.4% of all private schools in Denmark participate in regular outdoor teaching [13]. However, research results regarding those programmes are often only based on case studies using an arsenal of different methodological approaches.

Nevertheless, recent educational school reforms can be observed in several countries. The Danish reform “Improving the Public School” explicitly aims to increase PA during the school day; a longer school day with a special focus on learning, motivation and well-being; and working more closely with local sports clubs and cultural centres [14]. Recommendations to teach several curriculum content areas outside the classroom can be found in the new regional curriculum in Bavaria, Germany [15]. Furthermore, a shift towards multi-disciplinary, phenomenon- and project-based teaching was projected within the “National Core Curriculum 2016” in Finland [16,17]. Well-structured and curriculum-integrated outdoor education programmes could therefore offer great opportunities in helping to achieve the above-mentioned objectives.

In the last decades, six important reviews and meta-analyses in the field of outdoor education have been published [1,2,3,18,19,20]. Rickinson et al. [2], for example, set a wide focus on outdoor learning by evaluating the impact of: (i) fieldwork and visits; (ii) outdoor adventure activities; and (iii) school grounds and community projects. The authors summarised diverse benefits for each category, e.g., an increase in PA and academic achievement, development of social skills and a favourable attitude towards the environment. The recent systematic review of Fiennes et al. [20] partially updated the work of Rickinson et al. [2] by analysing primary research studies on outdoor learning from the UK that have been published since 2003. Similar to the conclusion of Rickinson et al. [2], most of the studies showed positive effects on a wide range of outcomes. The main study topics were still adventurous and residential activities while only a few studies were strongly linked to core curriculum subjects.

Only one review took a close look at the context of regular outdoor education within the school curriculum. Waite, Bølling and Bentsen [1] compared studies on Danish udeskole and English forest schools with a focus on purposes, aims, pedagogy, content, outcomes and barriers. The authors especially highlighted that both concepts seem to support children in their social and academic achievement, as well as their physiological and psychological well-being.

The existing reviews and meta-analysis in the wider field of outdoor education give a valuable overview on outdoor education research and practise. However, the literature shows a wide range in the intervention length, target and age groups, programme approaches, and the methodologies used. Three publications analysed programmes in the context of Outdoor Adventure Education/Outdoor Adventure Programming [3,18,19]. Two reviews set a very wide [2,20], and one review a narrow [1], focus on different OEPs within the school context. In addition, in most of the reviews the included primary studies are limited to selected countries. Only one review [20] used a systematic approach with respect to approved guidelines, i.e., the Reporting of Primary Empirical Research Studies in Education (REPOSE) Guidelines [21], and two reviews were not published in peer-reviewed journals [2,20].

Our purpose was to summarise studies on regular compulsory school- and curriculum-based outdoor education programmes for participants aged 5–18 that had been published in peer-reviewed journals. We aimed at: (i) categorising and evaluating reported outcomes; (ii) assessing the methodological quality of the included studies; and (iii) discussing possible benefits on students’ development by such programmes.

## 2. Methods

To identify and analyse the existing literature on regular compulsory school- and curriculum-based outdoor education programmes, we chose to endorse a systematic review approach. Systematic reviews in the context of education were, however, criticised by several authors [22,23,24]. It is concluded that one has be aware of the respective possibilities as well as limitations a systematic review can offer. Therefore, we see our work in relation to the model of education research developed by Andrews [22]. According to this model, we tried to summarise what is published and what methodological approaches were used, to identify the gaps and methodological shortcomings in the reviewed studies [22]. We conducted the systematic review in accordance with the preferred reporting items for systematic review and meta-analysis (PRISMA) guidelines [22]. The PRISMA guidelines are a well-accepted tool for systematic reviews and meta-analyses, they provide a valuable overview on how to structure the research process and help authors to account for transparency, validity and reproducibility.

### 2.1. Search Strategy

On 8 April 2016, we searched through the electronic PubMed, Scopus, Education Source, ERIC, Green File, PsycARTICLES, SPORTDiscus and SocINDEX databases for English and German language peer-reviewed journal articles. The search string included two components: “objective” and “setting”. Whereas “objective” represented relevant terms in respect of the synonyms for outdoor education programmes, “setting” described the defined educational environment. We used the following search terms for “objective” and “setting”:

Objective: “outdoor education”, “outdoor learning”, “outdoor teaching”, “learning outside the classroom”, “out-of-classroom”, “experiential learning”, “expeditionary learning”, “udeskole”, “uteskole”, “friluftsliv”, “forest school”, “nature school”, “environmental education”, “place-based education”, “Draußenschule”, and “Draussenschule”.

Setting: “school” and “curriculum”.

We used Boolean search operators, parentheses, search fields and asterisk according to the database specifications. Furthermore, we screened reference lists and citations of included articles to identify additional relevant studies.

For a detailed protocol and search strategy, please refer to our registered and published protocol under the International Prospective Register of Systematic Reviews (PROSPERO) Number: CRD42016033002. These documents are also available under Supplementary Materials.

### 2.2. Eligibility Criteria

We only included studies meeting the following eligibility criteria:

- All types of study designs (e.g., control group design, quasi-experimental design, and case studies);
- Any type of formal school- and curriculum-based outdoor education programme involving children and adolescents (5–18 years);
- Regular weekly or bi-weekly classes in a natural or cultural environment outside the classroom with at least four hours of compulsory educational activities per week over a period of at least two months; and
- At least one reported outcome on a student level.

No restrictions on publication periods were given.

### 2.3. Selection Process

Two independent reviewers (CB and GL) gradually screened all the titles and abstracts of studies identified for eligibility according to the criteria. Based on given information within the titles and abstracts, we made decisions about inclusion or exclusion. For studies that looked as if they would fulfil the inclusion criteria, we screened the full texts. If insufficient information was given in the abstract in order to make a clear exclusion decision, the full text was also screened. Any disagreements between reviewers were resolved by discussion. Both reviewers carefully documented their results after each step. We contacted the corresponding authors of 30 studies and requested additional information about the intervention and analyses procedures.

Both reviewers screened the reference lists and citations of included studies listed in Scopus using the same procedure to identify additional relevant studies.

### 2.4. Data Extraction

For each included study, we extracted data using a piloting form in respect to the required items. When essential information was not available from the full texts, we asked the corresponding authors to provide more information. Extracted data included:

1. Study characteristics: Citation, author, date of publication, journal, study-design, and country;
2. Population: Age, gender, sample size, and type of school;
3. Intervention characteristics: intervention and data acquisition period, and amount of intervention;
4. Methodology and analytic process.
5. Reported outcomes and main results.
6. Barriers and limitations.
7. Information for assessment of the risk of bias; and
8. Source(s) of research/project funding and potential conflicts of interest.

### 2.5. Analysis and Synthesis

Options for statistical quantitative analyses, including, risk ratios and standardised mean differences, were limited due to the heterogeneity of study designs, the range of measured outcomes and the overall small number of included studies. We therefore firstly provide a flow chart on the search and selection process and three tables presenting the main descriptive characteristics as well as the reported main outcomes of the included studies. Secondly, we qualitatively describe the most important outcomes of the studies in a narrative synthesis. Thirdly, we present results of the methodological quality assessment of included studies both in tables and narrative text.

### 2.6. Methodological Quality Assessment

Two reviewers (CB and GL) assessed the methodological quality of included studies. Additionally, one more independent reviewer (FM) had to specifically evaluate one article [23] which had been included in the review, due to the authorship of GL and UD who are part of the review team. Any disagreements between the reviewers were resolved through discussion and by referring to a third reviewer (UD). The quality of quantitative studies was appraised using the Child Care and Early Education Research Connections (CCEERC) Quantitative Research Assessment Tool [24]. The quality of qualitative studies was appraised using the Joanna Briggs Institute (JBI) Checklist for Qualitative Research [25]. Both tools were used for studies using quantitative, as well as qualitative, methods. For each tool, an overall rating was conducted based on the given assessment criteria. Quantitative studies were rated on 12 questions using a scale: 1, 0, −1, and n/a (not applicable); to account for completeness one question on research ethics was adapted by the JBI Checklist for Qualitative Research. Qualitative studies were rated on 9 questions using a scale: y (yes), n (no), u (unclear), and n/a (not applicable). One item was excluded due to inappropriateness within the research field. For further analyses, we adjusted the qualitative scale similar to the quantitative scale to the level of 1 (y), 0 (u), −1 (n), and n/a. For both quantitative and qualitative studies, an overall rating is presented in Appendix A Table A1 and Table A2 with mean values and standard deviations. Based on the mean values, we provide an overall rating regarding the categories low, moderate and high methodological quality. The cut-off values are defined as follows: low = M < 0.30; moderate = 0.30 ≤ M ≤ 0.60; and high = M > 0.60. They are based on theoretical assumptions in relation to methodological quality. Our approach, including the cut-off values based on the mean values, should be seen as a relative rating in relation to our data to provide a comparison of methodological quality. To our knowledge, no other rating system is available in relation to the applied tools. No studies were excluded from the review based on their methodological quality assessment results to ensure that all the potential valuable results are presented [26].

## 3. Results

**Figure 1:**
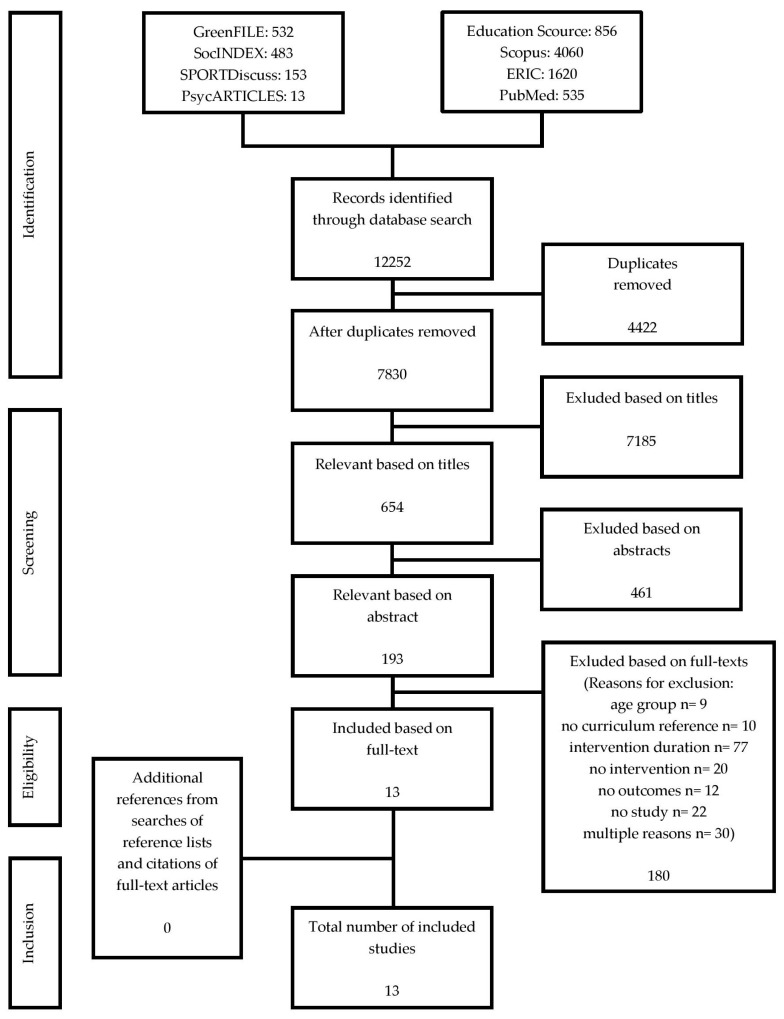
Flow chart of study search and selection process.

Figure 1 shows the selection process in general, numbers for each stage of the selection process and reasons for exclusion after screening the full papers. After the exclusion of direct duplicates, the literature search in the various databases yielded 7830 potentially relevant publications. After we screened titles and abstracts, we retrieved 193 studies in full-text. Thirteen studies met all the eligibility criteria. We looked at reference lists and citations of included studies listed in Scopus. Both the reference list search and the cited-by-search yielded no additional studies that met all the eligibility criteria. Finally, we included 13 studies in this systematic review.

### 3.1. Characteristics of Included Studies

Table 1 shows the main descriptive characteristics of the 13 included studies. Table 2 shows specific information concerning the interventions and data collections. Four studies were conducted in Denmark [4,6,7,27], three in the USA [28,29,30], and one each in Germany [23], New Zealand [31], Sweden [5], the UK [32], and Norway [33]. One study included data from the UK, India and Kenya [34]. The sample sizes varied considerably across the studies, from five [6] to 230 [5] children/adolescents being involved. Nine studies are defined as case studies [4,6,7,27,29,31,32,33,34], three studies used a quasi-experimental design [5,28,30], and one study used a cross-sectional design [23]. Three publications [4,6,7] are based on the same intervention, while all other publications are based on individual interventions. Six studies collected and analysed data on a solely student level [4,23,27,29,34,35], five studies also included data from teachers, staff and parents [6,30,31,32,33] and one study [5] only included data from parents. Eight studies used interviews [6,27,29,30,31,32,33,34], six studies used questionnaires [5,7,28,30,31], three studies used learning assessments [29,30,31], two studies used observations [32,34] and, in each case, one study used a postal survey [23], written documents [29], drawings and concept maps [34], and accelerometry [4]. The quantity of compulsory educational activities in a natural or cultural environment outside the classroom varied from one school day bi-weekly to a duration of eight weeks [27], and a six-month full week programme [23]. The chosen environments also differ between the studies: gardening projects on school grounds or nearby community properties [27,32,34], classes in a local forest [4,6,7,31,33], prairie [30] and farmland areas [29], the use of nearby school environments [5,28], and an overseas sailing expedition [23].

The included studies are very heterogeneous in respect of their study design, used methods and instruments, learning environments and measured outcomes. We categorised measured outcomes and presented the results of each study according to the study design. Seven studies reported outcomes on learning dimensions [7,27,29,30,31,32,34] and eight studies on social dimensions [6,7,23,27,28,30,31,34]. Two studies reported on students’ physical activity [4,7], one study [5] on students’ mental health, one study [33] on students’ action regulation behaviour and one study [31] on students’ environmental attitude and behaviour.

### 3.2. Methodological Quality Assessment of Included Studies

The methodological quality for most of the quantitative studies [4,7,28,30,31] can be classified as low (M = −0.14, SD = 0.31) to moderate (M = 0.50; SD = 0), with mean values ranged from −0.45 to 0.45; one study was rated as high, with a mean value of 0.67 (M = 0.67; SD = 0) [5]. Main reasons for the low or moderate ratings result from a poor description of the population of interest, the non-random selection of participants, insufficient presentation of means and standard variations/standard errors for numeric variables, the handling of missing data, the inappropriateness of statistical techniques and handling of alternative explanations, insufficient information according to current ethical criteria, and missing model coefficients and standard errors for main effect variables.

The methodological quality for most of the qualitative studies [6,27,29,30,31,32,33] can be classified as moderate (M = 0.41, SD = 0.12), with mean values ranged from 0.33 to 0.56. Two studies were rated as high (M = 0.78, SD = 0) [23,34], with mean values of 0.78 each. Main reasons for the low ratings result from insufficient information about the influence of the researcher on the observed or interviewed participants, and vice-versa; insufficient information according to current ethical criteria; and an inappropriate connection between the conclusions and the analyses.

A detailed description of the methodological quality assessment is presented in Appendix A Table A1 and Table A2.

### 3.3. Categorised Outcomes

We categorised the reported outcomes of studies on regular school and curriculum OEP. Table 3 shows the main outcomes in order to categorise students’ learning dimensions, social dimensions and additional outcomes.

#### 3.3.1. Outcomes on Learning Dimensions

Six case studies [7,27,29,31,32,34] analysed datasets concerning learning dimension. Mygind [4] conducted a study with primary school children attending a three-year outdoor education project. Students were asked about their perceptions on teaching and learning during indoor and outdoor classes by means of a questionnaire. Significant differences were found in three out of 14 statements: students liked the outdoor setting more than the indoor setting (*p* < 0.05), they were more careless about homework in the indoor setting (*p* < 0.01) and more disturbances in group work activities occurred during the indoor setting (*p* < 0.05). No significant differences were found for the other 11 statements.

Santelmann et al. [29] conducted a study with sixth- to eighth-grade students participating in a one-year place-based curriculum. The authors analysed documents written by students, and interviews conducted by students using a semi-quantitative content analysis. It is concluded that, through direct interaction with landowners, students developed a better understanding of decision-making in farm and forest enterprises, and received insights into the global interconnectedness of agricultural markets. The students’ learning benefit during outdoor lessons was especially mediated through hands-on learning and active participation. In a self-evaluation learning assessment, 75% of the students reported having gained new knowledge about farms, forests and wildlife refuges, and 25% developed better communication skills towards adults.

Moeed et al. [31] conducted a study with ninth and tenth-grade students in a school-led and community-supported environmental education project. A subgroup of tenth-grade students was pre- and post-tested on horticulture skills using a learning assessment. Eighty-five per cent of the students improved their grade skills for all four skill sets (preparing seeds for germination, pricking out, transplanting seedlings, planting) by 13%. The students were also able to transfer the gained skills into a different context.

Bowker et al. [34] carried out a study with primary and secondary school children participating in a one-year school gardening project. The authors used a qualitative content analysis to analyse concept maps, drawings, interview transcripts, and contextual observations. Based on these analyses, the authors stated that the gardening experiences can have a positive impact on students’ curriculum learning.

Sharpe [32] evaluated how a one-year community gardening programme can be beneficial for fifth-grade students in building confidence and being prepared for academic success. Sharpe used a qualitative content analysis to analyse semi-structured interviews, contextual observations and drawings. It is reported that the students had strong contextualised learning opportunities in mathematics, English and science, which allowed them to apply learned content to real-life situations.

Wistoft [27] carried out a study with primary school children attending a half-year community-led garden project. The author applied a qualitative content analysis to analyse interview transcripts and questionnaires. A summary of students’ learning dimensions yielded in three main categories: (i) learning through enjoyment and experiences; (ii) the ability to use knowledge, understanding and the skills acquired; and (iii) learning through the outdoor life. Students’ learning opportunities were made possible by the teachers’ passion and love for teaching. As a main conclusion, the students developed a desire to learn through participation in the programme, which can be seen as an indicator of positive learning motivation.

Ernst et al. [30] evaluated learning dimensions of a one-year out-of-school science programme for fifth-grade students. The authors used standardised assessment tests to compare students’ learning achievements in reading, writing and mathematics, and found significantly higher reading and writing scores for students within the intervention group (IG), compared to students within the control group (CG) (*p* = 0.03). No results were given for scores in mathematics. Based on the self-report questionnaire analysis, a positive and significant increase in the science process, problem-solving, technology skills, skills in working, and communication for students within IG compared to students within CG (*p* < 0.01) was found. Ninety-eight per cent of the parents from students within IG stated in a questionnaire that their children learned science, maths and writing better than they would have done in a normal school setting. Parents mentioned hands-on learning practise, interdisciplinary instructional strategy and real-world applications within outdoor teaching as the main conditions for this positive learning environment. Students within IG stated in interviews that they became more interested in school and learning through the outdoor teaching.

#### 3.3.2. Outcomes on Social Dimensions 

Six case studies [6,7,27,30,32,34] analysed datasets concerning social dimensions. Mygind [7] used a questionnaire to ask students about their social relations during teaching and during the breaks, comparing indoor and outdoor classes. Significant differences were found in two out of 10 statements: students liked the outdoor setting more than the indoor setting (*p* < 0.05) and it was noisier during the indoor setting (*p* < 0.05). No significant differences were found for the other eight statements, i.e., “I tease my classmates in the...” or “I try to assist my classmates in the…” Hartmeyer et al. [6] conducted a study with students and teachers seven years after the students had participated in a three-year primary school outdoor education project. In a qualitative content analysis, semi-structured interviews with students and teachers were analysed. In conclusion, six conditions influenced students’ social relations during their school years. In detail, the students improved their social relations and four conditions seem to have been important for that: “play”, “interaction”, “participation” and “pupil-centred tasks”. Furthermore, this improvement in social relations, enabled through the four conditions, positively influenced the pupils’ ability to “co-operate” and to “engage” in subsequent school years. Bowker et al. [34] concluded that students who had taken part in a one-year gardening project developed an overall sense of pride, excitement and high self-esteem. The gardening experience had a positive impact on students’ general school experience, which was interpreted as an association between gardening activities and self-esteem. Sharpe [32] reported that students developed trusting relationships and educationally-focused symbiotic relationships during the one-year project. Furthermore, the project fostered students’ growth in self-confidence and experiences leading to them taking active responsibility for the environment. Wistoft [27] reported that students developed social competencies through active participation in the gardening project: improved team-working and communication skills, improved social relatedness, and an understanding of the importance of taking responsibility and having respect for others’ work and property.

One quasi-experimental study [28] analysed datasets concerning social dimensions.

Martin et al. [28] conducted a study to research the effects of a 10-week expeditionary learning programme on seventh and eighth-grade students’ environmental virtue. Students completed questionnaires and the environmental virtue score decreased significantly for students’ in intervention group (IG) in four out of five domains of environmental virtue: courage (*p* = 0.006); temperance (*p* = 0.084); acceptance (*p* = 0.014); and humility (*p* = 0.009). For students in the control group (CG), the changes in environmental virtue score were not significant.

One cross-sectional study [23] analysed datasets on social dimensions. Dettweiler et al. [23] conducted a study with students who had participated in one of overall four six-month overseas learning expeditions. To evaluate the students’ social readjustment strategies, they were asked to write letters about their experiences after they returned from the expedition. In a mixed-method approach, the authors analysed students’ readjustment strategies. The time intervals between the return and the data collection were different for every expedition. The statements on readjustment strategies from the students being at home for eight months were most negative compared to the students having less or more time to readjust. Therefore, students can experience symptoms of a reverse culture shock after a long-term overseas expedition. However, the longer the students had time to readjust, the more positive they report on perceived programme effects. No gender differences were found.

One quasi-experimental study [30] analysed datasets on social dimensions. Ernst et al. [30] compared students’ attitudes towards a specific local natural environment. The attitudes towards the environment of students in the IG changed significantly compared to that of students in the CG (*p* = 0.02). One hundred per cent of the parents of students in the IG stated in a questionnaire that their children expressed a positive attitude towards outdoor teaching and 98% stated that the outdoor teaching fostered students’ excitement about school in general. Students in the IG stated in interviews that their social behaviour had improved and that the outdoor lessons had advanced their social relatedness.

#### 3.3.3. Additional Outcomes

In addition to the aforementioned two categories of Outcomes on Learning and Social Dimensions, we clustered five studies [4,5,7,31,33] with specific outcomes under additional outcomes as they do not fit precisely into any other category.

Two case studies [4,7] analysed datasets on students’ physical activity. Mygind [4,7] conducted the studies with students participating in a three-year outdoor education project and measured their PA during outdoor and indoor learning. Objectively-measured PA was significantly higher during one outdoor learning day, compared to one traditional indoor school day, in 2000 and 2001 (both *p* < 0.001), while no significant differences in PA was found for one outdoor learning day, compared to one normal school day including two physical education lessons, in 2002 (*p* = 0.52) [4]. Students were asked, by means of a questionnaire, about their perceived physical activity. Students reported to have used their body significantly more often during classes in the outdoor teaching setting (*p* < 0.01) compared to the indoor setting and also to have been more active during the breaks in the outdoor setting (*p* < 0.01) compared to the indoor setting [7].

One case study [31] analysed datasets regarding students’ environmental attitude and behaviour. Moeed et al. [31] applied a qualitative content analysis to analyse interview transcripts. The authors conducted the interviews six or eight years respectively, after the students participated in an environmental project. The students showed a strong awareness of environmental issues and were actively involved in environmental community projects. The students traced both aspects back to their participation and experiences in outdoor classes.

One case study [33] analysed datasets with respect to students’ action-regulation behaviour. Fiskum et al. [33] conducted a study with fifth-grade primary school students who participated in outdoor classes over the period of five years. In a qualitative content analysis group interviews were analysed with a special focus on interaction between affordances, action-regulation, and learning. The authors reported that outdoor learning environments can offer a great variability in children’s choices of activity during classes. The main results relate to gender differences. Boys mainly grasped affordances specific to the outdoor environment and used their own creativity, whereas girls mainly grasped affordances not specific to the outdoor environment and used attached objects especially designed for them. Girls more often regulated their action in the outdoor setting compared to boys. Both girls and boys reported on several learning contents related to grasped affordances. It has been concluded that outdoor education compared to teacher-directed learning in the classroom, may provide better opportunities to reach the third level of cognitive process dimension—apply—by enabling conscious relationships concerning content and objects.

One quasi-experimental study [5] analysed datasets on students’ mental health status. Gustafsson et al. [5] conducted a study with primary school children who had attended an outdoor education project over a period of one school year. In a questionnaire, the parents stated their observations regarding their children’s psychiatric symptoms. When adjusted for demographics, no significant overall effect on mental health was found for students in the IG, compared to students in the CG with respect to total difficulties, as well as all the subscales (all *ps* > 0.1). However, a gender effect of the intervention was found. Mental problems significantly decreased for boys compared to girls, with respect to total difficulties (*p* < 0.001), as well as the subscales of “emotional symptoms” (*p* = 0.044), “conduct problems” (*p* < 0.003), and “hyperactivity” (*p* = 0.005). Effects were not significant for peer problems and pro-social behaviour.

## 4. Discussion

We aimed at systematically reviewing the current state of research on regular compulsory school- and curriculum-based outdoor education programmes. Specifically, we categorised and evaluated reported outcomes of 13 included studies and rated their methodological quality.

### 4.1. General Aspects

The current state of research is relatively small with only 13 identified and evaluated studies. This can partly be explained by the fact that outdoor education research is quite a young field of research, although, with a rising number of publications within the last years. The small number of included studies can also be attributed to the fact that efforts to conduct regular curriculum-based outdoor teaching face many barriers. Waite, Bølling and Bentsen [1] summarised the cost of transportation and extra teachers, travel-time, a crowded curriculum and teacher qualifications as main obstacles for more outdoor learning projects in schools in the UK and in Denmark.

We also applied certain inclusion criteria, such as a minimum intervention length of eight weeks. By further opening-up these criteria, more studies could naturally have been evaluated, but this would have simultaneously led to a renunciation of the comparability of the assessed studies and outcomes. Waite, Bølling and Bentsen [1], for example, therefore chose different inclusion criteria—less strict concerning, e.g., age group, intervention duration, publication type—and thus compared 39 similar studies concerning school-based outdoor education programmes. Compared to the related field of Outdoor Adventure Education/Outdoor Adventure Programming, the aforementioned literature reviews and meta-analyses reviewed several studies, e.g., 96 studies regarding the overall effects of adventure programmes [3], and 43 studies concerning outdoor adventure programmes for adolescents [19]. This can also be seen as an indication that more studies on regular compulsory school- and curriculum-based outdoor education programmes are needed, in order to gain a deeper understanding of the possible benefits.

### 4.2. Methodological Quality Assessment

The methodological quality assessment for most of the studies yielded moderate results.

Particularly, those results of studies with moderate or low methodological quality have to therefore be considered with caution. Apart from that, some important specific circumstances regarding the included studies have to be considered. Due to the nature of educational interventions, not all requirements for preventing possible methodological bias (e.g., randomisation, a high number of participants) can be fulfilled in practice and we applied two relatively strict assessment tools. In contrast to most natural science domains, formal ethical approvals are still not obligatory in some educational and sociological domains. Furthermore, official ethic committees still have to be established to a certain extent. Another explanation could be that researchers are incidentally unaware of the importance of such formal ethical issues. Furthermore, the aim of most (case) studies included in this systematic review was rather to explore the field and to describe specific (rare) cases, instead of giving the opportunity to generalise the results gained to a wider population. As mentioned above, several studies do show a lack of methodological quality. Although the methodological quality of research studies is not the main focus of this review—and one should not overestimate it when considering the possibilities of conducting studies in educational settings—these ratings can be seen as indicators for detecting shortcomings in this particular scientific field, and this is in concordance with results of the review by Scrutton et al. [18]. The authors examined studies in the related field of Outdoor Adventure Education, focusing on personal and social development. They stated that, frequently, the sample sizes used were too small, and went on to discuss the questionable usage and handling of questionnaires, as well as the statistical management of variables. Scrutton and colleagues [18] requested that future research should be carefully designed with regard to methodological rigour if the researchers’ aim is to actually inform and change educational policy.

Certain results must therefore be interpreted with respect to the study design used and its corresponding possibilities and weaknesses as regarding generalisability, validity and reliability.

### 4.3. Learning Dimensions

The presented results in the category of learning dimension, reported by seven studies [7,27,29,30,31,32,34], illustrate one main focus of the current research in the field of regular compulsory school- and curriculum-based outdoor education programmes.

According to the results on learning dimensions, students particularly seem to benefit in terms of an improved academic performance in several subjects, improved skills in transferring the knowledge gained to real life situations. In addition, two studies [27,34] mentioned possible benefits on aspects of students’ learning motivation, i.e., learning as fun and a desire to learn. Considering that learning motivation can be an important factor for academic success [35], and some studies in outdoor education settings [36,37,38] have already analysed motivational aspects of short-term interventions, this could possibly be a promising approach for future research.

The methodological quality for studies reporting on learning dimensions, however, is rated as moderate [7,27,29,30,32] except for one study which is rated as low [31]. Due to the methodological weaknesses, the reported results have to be considered with caution. However, they are in concordance with different literature reviews and meta-analyses concerning general outdoor education. Waite, Bølling and Bentsen [1] mentioned that regular udeskole enhances learning outcomes. Rickinson et al. [2] highlighted the benefits of school grounds/community projects on students’ science process skills as well as the impact of fieldwork and visits on students’ long-term memory and higher order learning. Furthermore, Cason and Gillis [19] found an average effect size of 0.61 (*n* = 10; SD = 1.527) of outdoor adventure programmes on adolescents’ grades and Hattie et al. [3] mentioned that “adventure programs enhance general problem solving competencies”, understood as a subcategory of academic performance (ES = 0.45; *n* = 23; CI = 0.23 to 0.67).

Taking into account these indications and respective methodological shortcomings, more high quality-studies are needed to further examine possible effects of regular outdoor classes on students learning dimensions.

### 4.4. Social Dimensions

The presented results in the category of social dimension, reported by nine studies [6,7,23,28,30,31,32,34], illustrates another main focus of the current research regarding regular compulsory school- and curriculum-based outdoor education programmes.

According to the results in social dimensions, students seem to benefit in terms of their development of social competencies and social relations such as self-esteem, self-confidence, trusting relationships, and the sense of belonging [6,7,27,30,32,34]. One study [23] also reported that students mentioned perceived positive programmes effects, however, with a temporal shift of approximately eight months. Furthermore, three studies reported positive effects on students’ attitudes and behaviour patterns towards the environment [30,31,32]. One study [28] mentioned negative effects on students’ environmental attitudes. The methodological quality for studies reporting on social dimension is rated as moderate [6,7,27,30,31,32] except for two studies rated as high [23,34]. Despite the methodological weaknesses, the reported results are in concordance with conclusions by Waite, Bølling and Bentsen [1]: Forest schools, as well as udeskole programmes, can promote students’ social relations, interpersonal skills, and social competencies. Furthermore, Rickinson et al. [2,5] summarised that fieldwork and visits “can lead to individual growth and improvements in social skills (…) and improve attitudes towards the environment” while school grounds and community projects can foster students’ sense of belonging, relationships and community involvement.

Similar to our demands regarding learning dimensions, there is also a strong need for more high quality-studies to further examine possible effects of regular outdoor classes on students’ social dimensions.

### 4.5. Additional Dimensions

The research on students’ physical activity, mental health and action regulation behaviour is underrepresented in comparison to results on students’ learning and social dimensions. Only two case studies [4,7] with moderate to low methodological quality, reported positive effects on students’ PA. Only one case study [33] with moderate methodological quality mentioned gender differences with respect to action regulation behaviour. Furthermore, only one quasi-experimental study [5], with a high methodological quality reported positive effects of regular outdoor classes on boys’ mental health. Therefore, the presented results of PA, mental health and action regulation behaviour can at most be interpreted as first indications. However, taking results from related publications into account, these indications can be partly supported. In detail, Rickinson et al. [2] showed in their review that school grounds and community projects can be beneficial for children’s exercise. Additionally, Waite, Bølling and Bentsen [1] mentioned that forest school and udeskole projects increased students’ PA and motor-skills. Regarding students’ mental health, Cason and Gillis [19] found an average effect size of 1.047 (*n* = 12; SD = 0.459) for adolescents’ clinical scales (e.g., depression and anxiety) regarding outdoor adventure programming.

More high quality-studies are therefore needed to further examine these first indications of the effects of regular outdoor classes on students’ PA, mental health and action regulation behaviour, especially when considering an increasing inactivity [39], as well as a rising number of diagnosed mental health disorders in school children [40].

### 4.6. Strengths and Limitations

There are four main strengths in this systematic review. First, we strictly referred to a search protocol and design according to the PRISMA Guidelines and applied several online databases for literature research. Secondly, the chosen inclusion criteria allowed for the consideration of a wide range of studies concerning study design, country, target group and reported outcomes. Thirdly, two reviewers independently screened the literature and assessed the methodological quality of the included studies and, fourthly, we applied the CCEERC Quantitative Research Assessment Tool as well as the JBI Checklist for Qualitative Research to rate the studies’ methodological quality.

However, we only evaluated studies published in English and German in peer-reviewed journals and listed in the used online databases, but no grey literature or reports. We therefore cannot rule out the existence of relevant studies in other languages or studies published elsewhere. Furthermore, we observed that several included, as well as excluded, articles were weak in respect of the internal structure and given information. Hypothesising that this is a wide spread practice, this could also mean that other valuable research results had not been properly published in peer-reviewed journals, and were therefore not eligible for inclusion in this systematic review.

These limitations are in concordance with the critique on systematic reviews in education, as described in the methods chapter. Therefore, we cannot claim to have delivered an all-embracing solution to the questions we have asked. We have not “eliminate(ed) bias” nor have we “present(ed) an ‘objective’ version of the truth, but” we have “attempt(ed) to minimise bias” in the field [41].

## 5. Conclusions

To conclude, the number of identified studies on regular compulsory school- and curriculum-based outdoor education programmes is relatively low. In addition, these 13 evaluated studies show wide heterogeneity in respect of the aims, participant groups, learning environments, methods used and reported effects, and the methodological quality is, on average, moderate. However, tendencies were found which indicate that regular compulsory school- and curriculum-based outdoor education programmes can advance students in the physical, psychological, learning and social dimensions.

To further evaluate these indications, more research studies are needed. Thereby, a strong focus on aspects of study design and methodological quality has to be set. Especially randomised-controlled trials, longitudinal studies and studies that are more quasi-experimental with a higher number of participants are desirable for future research. Additionally, the intervention duration should be as long as possible, as it has been shown that longer programmes lead to better effects [2]. Future research should particularly focus on aspects of students’ PA and mental health, as we have shown that those are underrepresented in the reviewed literature.

However, these study designs are often difficult to conduct in educational settings, especially as practical “Outdoor Education’ strongly depends on the respective teachers” motivation and beliefs, their pedagogical concepts and ideas, and a certain financial support from headmasters/headmistresses and school authorities [1,12]. If practitioners, researchers and policymakers work more closely together in a dialogic relationship and with a strong focus on what is needed, as demanded by Fiennes et al. [20] and Andrews [41], positive changes in school practise can hopefully be realised for students’ benefits. This can partly be seen in relationship to a recent OECD report on learning environments in the 21st century. According to the report, innovative learning environments are needed. Specifically, a combination of pedagogical approaches on “guided learning”, “action learning” and “experiential learning” that enables self-regulated learning [42]. Although not being the focus in our review, the underlying pedagogical concepts in outdoor education do set focus at least partially on these learning environments [1].

One promising example is the Danish TEACHOUT research project which used a quasi-experimental and longitudinal design to analyse the impacts of regular outdoor teaching on 834 students’ PA, well-being, social interaction and learning [43]. First results are to be expected in 2017. In the future, more such high-quality studies should be realised by referring to a rich theoretical background and methodology, as well as informing and including policy and school administration.

## Figures and Tables

**Table 1:** Descriptive characteristics of studies on regular school- and curriculum-based outdoor education programmes.

Source	N	Age	Distribution of Sex (% Male)	Country	Study Design	Administrator of Data Acquisition	Type of School
Mygind [4]	19	9–10	26.3	Denmark	case-study	chn	primary school
Mygind [7]	19	9–10	26.3	Denmark	case-study	chn	primary school
Dettweiler et al. [23]	56	14–20	n/a	Germany	cross-sectional retrospective	adol	secondary school
Hartmeyer et al. [6]	5 ^adol^, 2 ^t^	16	40 ^adol^	Denmark	case-study retrospective	adol, t	primary school
Martin et al. [28]	45 ^IG^, 67 ^CG^	14–15	51.1 ^IG^, 47.8 ^CG^	USA	quasi-experimental	adol	secondary school
Santelmann et al. [29]	40	12–15	n/a	USA	case-study	chn, adol	secondary school
Moeed et al. [31]	85 ^adol^, 1 ^t^	15-24	61 ^adol^	New Zealand	case-study	adol, adul, t	secondary school
Gustafsson et al. [5]	121 ^IG^, 109 ^CG^	8.6 ± 1.6 ^IG^, 8.1 ± 1.5 ^CG^	56.2 ^IG^, 51.4 ^CG^	Sweden	quasi-experimental	chn	primary school
Bowker et al. [34]	72	7–14	n/a	UK, India, Kenya	case-study	chn, adol	primary + secondary school
Sharpe [32]	9 ^chn^, 2 ^t^, 5 ^p^, 2 ^s^	10–11	n/a	UK	case-study	chn, t, p, s	primary school
Fiskum et al. [33]	9	10–11	55.6	Norway	case-study	chn	primary school
Wistoft [27]	98 ^chn/t^, 135 ^p^, 6 ^s^	-	n/a	Denmark	case-study	chn, p, t, s	primary school
Ernst et al. [30]	90 ^chn^, n/a ^p s^	10–11 ^chn^	n/a	USA	quasi-experimental	chn, p, s	secondary school

Note: adol: adolescents; chn: children; p: parents; t: teacher; s: staff; IG: intervention group; CG: control group; n/a: not available.

**Table 2:** Characteristics of intervention and data collection of studies on regular school- and curriculum-based outdoor education programmes.

Source	Data Collection	Intervention Period and Data Acquisition	Intervention Length
Mygind [4]	objectively-measured physical activity; accelerometry devise: CSA 7164 activity monitor	IP: school years 2000/2001/2002 DA: school years 2000/2001/2002	three school years; one outdoor school day each week
Mygind [7]	adapted version of “About my self—a questionnaire for children” on self-perceived physical activity level, social relations and learning behaviour	IP: school years 2000/2001/2002/2003 DA: school years 2000/2001/2002/2003	three school years; one outdoor school day each week
Dettweiler et al. [23]	postal survey; hand written letter	IP: 2008/2009/2010/2011 DA: 2012	six months; each expedition
Hartmeyer et al. [6]	semi-structured interviews	IP: school years 2000/2001/2002/2003 DA: 2010	three school years; one outdoor school day each week
Martin et al. [28]	Children’s Environmental Virtue Scale (CEVS) Questionnaire, adapted and modified by Children’s Environmental Attitude and Knowledge Scale (CHEAKS)	IP: 10/2005-01/2006 DA: 10/2005+01/2006 ^IG^; spring semester 2006 ^CG^	10 weeks; at least one half day per week
Santelmann et al. [29]	interviews, written documents, learning assessment	IP: school year 2006/2007 DA: 2006/2007	one school year; one outdoor school day in 1/3 of all weeks
Moeed et al. [31]	unspecified self-evaluation questionnaire, interviews, learning assessment	IP: 1997–1998 DA: 1997–1998	two school years; four hours bi-weekly year nine; four hours weekly year 10
Gustafsson et al. [5]	Strengths and Difficulties Questionnaire (SDQ), parent-version	IP: school year 2002/2003 DA: autumn 2002/autumn 2003	one school year; five days per week; at least one hour per day
Bowker et al. [34]	concept maps, semi-structured group interviews, contextual observations, drawings	IP: school year 2004/2005 DA: school year 2004/2005	one school year; four hours on average each week
Sharpe [32]	semi-structured individual interviews, group interview, observations	IP: school year 2012/2013 DA: summer/autumn 2013	one school year; four hours on average each week
Fiskum et al. [33]	group interviews	IP: school years 2004–2008 DA: autumn 2008/spring 2009	five school years; one outdoor day per week, years 1–4, one outdoor school day bi-weekly, year five
Wistoft [27]	group interviews, individual interviews, unspecified questionnaires	IP: school year 2010/2011 DA: school year 2010/2011	eight weeks; one outdoor school day bi-weekly; 7–8 h on average per day
Ernst et al. [30]	Skills Self-Report questionnaire; Affective Self-Report and Parent Survey questionnaire, both developed by the author; standardised assessment test: Minnesota Comprehensive Assessments in Maths and Writing; individual interviews	IP: school year 2003/2004 DA: school year 2003/2004	one school year; five days per week; two hours per day

Note: IG: intervention group; CG: control group; IP: intervention period; DA: data acquisition.

**Table 3:** Reported outcomes of studies on regular school- and curriculum-based outdoor education programmes.

Source	Outcomes on Learning Dimensions	Outcomes on Social Dimensions	Additional Outcomes
Mygind [4]			PA significant higher during outdoor classes compared to indoor classes (*p* < 0.001, 2000/2001); no significant differences in PA between outdoor classes and indoor classes including 2 PE lessons (*p* = 0.52, 2002); significant −level: 0.05
Mygind [7]	higher preferences for learning in the outdoor setting compared to indoor setting; significant differences in three out of 14 statements	significant more positive social relations in the outdoor setting compared to the indoor setting (*p* < 0.001); significance-level: 0.05	significant higher perceived PA in the outdoor setting (*p* < 0.01); significant −level: 0.05
Dettweiler et al. [23]		long-term educational overseas expedition can lead to symptoms of a reverse culture shock; similar readjustment problems and development of coping strategies for all the participants, shown in a U-curve model; the longer the students had time to readjust, the more positive they report on perceived programme effects, shown as a linear function; no differences between cruises and gender	
Hartmeyer et al. [6]		identification of six important conditions for the improvement of social relations: play, interaction, participation and pupil-centred tasks—important for positive social relations during udeskole; co-operation and engagement—consequences of improved social relations in subsequent years	
Martin et al. [28]		IG: significant decrease in 5 CEVS domains: courage (*p* < 0.006); temperance (*p* = 0.084); acceptance (*p* = 0.014); compassion (*p* = 0.109); humility (*p* = 0.009); CG: significant decrease in courage (*p* = 0.169) and increase in temperance (*p* = 0.389); acceptance (*p* = 0.553); compassion (*p* = 0.796); humility (*p* = 0.553); significance-level: 0.1	
Santelmann et al. [29]	improved understanding of decision-making on farm and forest enterprises; insights into the global interconnectedness and ecodynamic drivers of agricultural markets		
Moeed et al. [31]	year 10 students: improved horticulture skills (85% improved grade with 13%); year 9 students: strong level of commitment to develop knowledge and skills		former students: long term effects of the programme concerning positive environmental behaviour: growing own vegetables, participating in community-based planting programmes, taking own students outdoors within environmental projects, cleaning the Himalayas
Gustafsson et al. [5]			overall positive, but not significant effect on mental health in the IG (*p* > 0.1); significant decrease in mental health problems for boys in IG compared to CG (*p* < 0.001); no significant differences for girls; significance-level: 0.1
Bowker et al. [34]	gardening experience has a positive impact on curriculum learning: indication of direct association between gardening activities and improved learning	overall sense of pride, excitement and high self-esteem; gardening experience had a positive impact on students’ general school experience: indication of direct association between gardening activities and self-esteem	
Sharpe [31]	strong contextualised learning opportunities for children in Maths, English and Science; learning is perceived as fun through imaginative and creative learning opportunities; transfer from the indoor and outdoor classroom to real-life situations	building of trusting relationships and educationally-focused symbiotic relationships; growth in self-confidence; experience to take active responsibility for the environment	
Fiskum et al. [33]			gender differences: boys more often grasped affordances specific to the outdoor environment and used own creativity; girls more often grasped affordances not specific to the outdoor environment and used attached objects especially designed for them; girls more often regulate their action in the outdoor setting
Wistoft [27]	students developed a desire to learn through participation in the programme; they learned through enjoyment and experiences, they perceived learning as fun	students developed social competencies through participation in the programme	
Ernst et al. [30]	significant higher reading + writing scores for IG compared to CG (*p* = 0.03); positive significant increase in science process, problem-solving, technology skills, skills in working and communication for IG compared to CG (*p* < 0.01); students in the IG became more interested in school and learning fostered by outdoor learning	positive significant difference in students' attitudes towards the prairie wetlands environment for IG compared to CG (*p* = 0.02); IG students improved their classroom behaviour and prompted a sense of belonging	

Note: IG: intervention group; CG: control group, PA: physical activity; sig: significant; PE: physical education.

## Supplementary Materials

The following are available online at www.mdpi.com/1660-4601/14/5/485/s1, PROSPERO Protocol and Search Strategy, Reference Number CRD42016033002.; adapted versions of the CCEERC Quantitative Research Assessment Tool; and the JBI Checklist for Qualitative Research.

## Appendix A

**Table A1:** Methodological quality assessment for quantitative studies.

Source	Q1	Q2	Q3	Q4	Q5	Q6	Q7	Q8	Q8	Q10	Q11	Q12	Mean	SD
Mygind [4]	0	0	0	1	1	1	−1	0	−1	1	−1	−1	0.00	0.00
Mygind [27]	0	0	0	1	1	1	0	0	−1	1	−1	−1	0.08	0.79
Martin et al. [29]	0	0	1	1	1	1	0	0	0	1	0	1	0.50	0.52
Moeed et al. [32]	0	0	1	1	−1	−1	−1	−1	−1	−1	−1	−1	−0.50	0.80
Gustafsson et al. [5]	0	0	1	0	1	1	1	0	1	1	1	1	0.66	0.49
Ernst et al. [31]	0	0	1	1	1	1	0	0	0	1	0	−1	0.33	0.65

SD: standard deviation.

**Table A2:** Methodological quality assessment for qualitative studies.

Source	Q1	Q2	Q3	Q4	Q5	Q7	Q8	Q9	Q10	Mean	SD
Dettweiler et al. [23]	1	1	1	1	1	−1	1	1	1	0.78	0.67
Hartmeyer et al. [6]	1	1	1	1	1	−1	1	1	−1	0.56	0.88
Santelmann et al. [30]	0	0	1	1	1	−1	1	−1	1	0.33	0.86
Moeed et al. [32]	1	1	1	−1	1	−1	1	−1	1	0.33	1
Bowker et al. [35]	1	1	1	1	1	−1	1	1	1	0.78	0.67
Sharpe [33]	1	1	1	1	1	−1	1	0	−1	0.44	0.88
Fiskum et al. [34]	0	0	1	1	1	1	1	−1	−1	0.33	0.87
Wistoft [28]	1	1	1	1	1	−1	1	−1	1	0.56	0.88
Ernst et al. [31]	1	1	1	1	1	−1	1	−1	1	0.56	0.88

SD: standard deviation.
