# Supplementary material for: Effects of Regular Classes in Outdoor Education Settings: A Systematic Review on Students’ Learning, Social and Health Dimensions
Source: Int J Environ Res Public Health. 2017 May 5;14(5):485. doi: 10.3390/ijerph14050485 (PMC5451936; doi:10.3390/ijerph14050485)
Supplement: Supplementary file 1 [file ijerph-14-00485-s001.pdf]

Quantitative Research Assessment Tool

The purpose of this assessment tool is to help users quickly evaluate the merit of quantitative research studies that are listed on the CCEERC Web site. The assessment tool provides guidelines on two issues: 1) information that should be included in study reports, and 2) standards for good research. This tool should be used for general guidance when assessing the merit of research studies. For some child care and early education research, it may be difficult to implement the most rigorous research designs. When comparing scores derived through this assessment tool, studies with lower scores -- especially when examining research exploring the same questions -- should be viewed with more caution than studies with higher scores. Studies that do not include sufficient information to answer the questions asked in the assessment tool should also be viewed with caution.

Population and Sample

1. Population. Does the population that was eligible to be selected for the study include the entire population of interest? Or, is the eligible population a selective subgroup of the population of interest? For example, are all the children in the nation eligible to be selected for the study (the entire population of interest)? Or, were only children in New York City eligible to be selected for the study (a selective subgroup)? Or, were only children in one day care center in New York City eligible to be selected (a very selective subgroup)?

[1] Eligible population includes entire population of interest or a substantial portion of
it [0] Population represents a limited, atypical, or selective subgroup of the population
of interest
[-1] No description of the population
[NA] Not applicable

2. Randomized Selection of Participants. Were study participants randomly selected for the study? Or, did study participants volunteer (nonrandom)? Or, were they located through specific organizations (nonrandom) or through acquaintances of the researchers (nonrandom)?

[1] Random selection
[0] Nonrandom selection
[-1] No description of the sample selection
procedure [NA] Not applicable

3. Sample Size. How many participants were selected for the study? Does the sample include enough participants from key subgroups to accurately assess subgroup differences? This is best used in comparison to other studies.

[1] Sample size larger than similar studies
[0] Sample size the same as similar studies
[-1] Sample size smaller than similar study or sample size not given
[NA] Not applicable

4. Response and Attrition Rate. What proportion of the selected sample completed the study? In longitudinal studies, what proportion of sample members participated in follow-up studies?

[1] High response or participation rate (over 65% response rate, over 90% participated in follow-up studies)
[0] Moderate to low response rate (response rates of less than 65%)
[-1] No information on response rate or participation rate
[NA] Not applicable

Measurement

5. Main Variables or Concepts. Are each of the main variables or concepts of interest described fully? Can the main variables or concepts be matched to the variables in the tables?

[1] Accurately described and can be matched
[0] Vague definition or cannot be matched
[-1] No definition of main variables or concepts
[NA] Not applicable

6. Operationalization of Concepts. Did the authors choose variables that make sense as good measures of the main concepts in the study? Have these variables been used in previous studies or are they an improvement over previous studies?

[1] Key concepts are measured with variables that make sense. Or, variables have either been previously used in research or are improvements over previous measures.
[0] Key concepts are measured with variables that do not make sense, and variables have not been used in previous research studies
[-1] Variable operationalization is not discussed [NA] Not applicable

Analysis

7. Numeric Tables. Are the means and standard deviations/standard errors for all the numeric variables presented?

[1] Means and standard deviations/standard errors presented
[0] Means, but no standard deviations/standard errors presented
[-1] Neither means nor standard deviations/standard errors presented
[NA] Not applicable

8. Missing Data. Are the number of cases with missing data specified? Is the statistical procedure(s) for handling missing data described?

[1] Number of cases with missing data are specified and the strategy for handling missing data is described
[0] Number of cases with missing data specified, but these cases are removed from the

analysis
[-1] Missing data issues not discussed
[NA] Not applicable

9. Appropriateness of Statistical Techniques. Does the study describe the statistical technique used? Does the study explain why the statistical technique was chosen? Does the study include caveats about the conclusions that are based on the statistical technique?

[1] Statistical techniques, reasons for choosing technique, and caveats are fully explained
[0] Statistical technique is explained, but the reasons for choosing technique or the caveats are not included.
[-1] Statistical technique, reasons for choosing technique, and caveats are not explained. [NA] Not applicable

10. Omitted Variable Bias. Could the results of the study be due to alternative explanations that are not addressed in the study?

[1] All important explanations are included in the analysis [
0] Important explanations are omitted from the analysis
[-1] Variables and concepts included in the analysis are not described in sufficient detail to determine whether key alternative explanations have been omitted
[NA] Not applicable

11. Analysis of Main Effect Variables. Are coefficients for the main effect variables in the statistical models presented? Are the standard errors of these coefficients presented? Are significance levels or the results of statistical tests presented?

[1] Model coefficients and standard errors or hypothesis tests for the main effects variables are presented
[0] Either model coefficients or hypothesis tests for the main effects variables are presented [-1
] Neither estimated coefficients or standard errors for the main effects variables are presented
[NA] Not applicable

12. Is the research ethical according to current criteria or, for recent studies, and is there evidence of ethical approval by an appropriate body?

[1] Statements according to ethical criteria are appropriate presented by, i.e. statements on written consent of participants/parents, ethical approval of Universities Review Board, confidentiality of participants' identities.

[0] Statements according to ethical criteria or an ethical approval are incomplete.

[-1] No statements according to ethical criteria or an ethical approval are presented
